# Functional properties of the HIV-1 long terminal repeat containing single-nucleotide polymorphisms in Sp site III and CCAAT/enhancer binding protein site I

**DOI:** 10.1186/1743-422X-11-92

**Published:** 2014-05-16

**Authors:** Sonia Shah, Aikaterini Alexaki, Vanessa Pirrone, Satinder Dahiya, Michael R Nonnemacher, Brian Wigdahl

**Affiliations:** 1 Department of Microbiology and Immunology, and Center for Molecular Virology and Translational Neuroscience, Institute for Molecular Medicine and Infectious Disease, Drexel University College of Medicine, Philadelphia, Pennsylvania 19102, USA

**Keywords:** HIV-1 genetics, Viral transcription, Integration, Viral latency, Single-nucleotide polymorphisms, C/EBP, Sp

## Abstract

**Background:**

HIV-1 gene expression is driven by the long terminal repeat (LTR), which contains many binding sites shown to interact with an array of host and viral factors. Selective pressures within the host as well as the low fidelity of reverse transcriptase lead to changes in the relative prevalence of genetic variants within the HIV-1 genome, including the LTR, resulting in viral quasispecies that can be differentially regulated and can potentially establish niches within specific cell types and tissues.

**Methods:**

Utilizing flow cytometry and electromobility shift assays, specific single-nucleotide sequence polymorphisms (SNPs) were shown to alter both the phenotype of LTR-driven transcription and reactivation. Additional studies also demonstrated differential loading of transcription factors to probes derived from the double-variant LTR as compared to probes from the wild type.

**Results:**

This study has identified specific SNPs within CCAAT/enhancer binding protein (C/EBP) site I and Sp site III (3 T, C-to-T change at position 3, and 5 T, C-to-T change at position 5 of the binding site, respectively) that alter LTR-driven gene transcription and may alter the course of viral latency and reactivation. The HIV-1 LAI LTRs containing the SNPs of interest were coupled to a plasmid encoding green fluorescent protein (GFP), and polyclonal HIV-1 LTR-GFP stable cell lines utilizing bone marrow progenitor, T, and monocytic cell lines were constructed and utilized to explore the LTR phenotype associated with these genotypic changes.

**Conclusions:**

Although the 3 T and 5 T SNPs have been shown to be low-affinity binding sites, the fact that they can still result in effective HIV-1 LTR-driven gene expression, particularly within the TF-1 cell line, has suggested that the low binding site affinities associated with the 3 T C/EBP site I and 5 T Sp site III are potentially compensated for by the interaction of nuclear factor-κB with its corresponding binding sites under selected physiological and cellular conditions. Additionally, tumor necrosis factor-α and Tat can enhance basal transcription of each SNP-specific HIV-1 LTR; however, differential regulation of the LTR is both SNP- and cell type-specific.

## Background

HIV-1-associated immunologic and neurologic disease is dependent on the ability of the virus to infect subsets of resident immune and central nervous system (CNS) cell populations. In vitro and in vivo investigations have shown that HIV-1 infection of active CD4^+^ T lymphocytes initiates a highly productive infection [1-7]. In contrast, HIV-1-infected monocytic cell populations produce only limited quantities of virus due to several host-cell replication blocks including barriers that limit the reverse transcription process [8,9] and nuclear import [10]. These barriers result in a more chronic infection because this cell type is more resistant to the cytopathic effects of HIV-1 gene products [11-13] and has a longer lifespan in vivo. The chronic nature of HIV-1 replication in cells of the monocyte-macrophage lineage is likely a contributor to the central importance of these cells in evasion of HIV-1 detection and elimination by the immune system and the maintenance of viral reservoirs. The virus can utilize cells of this lineage as a vehicle facilitating its transport across the blood–brain barrier (BBB) and its entry into the CNS [14-16], thereby promoting HIV-1-associated neuropathogenesis and the development of minor neurocognitive impairment, as well as the more severe CNS disease, HIV-1-associated dementia (HIVD). HIV-1 infection of the CNS occurs soon after infection; however, under most circumstances, prolonged productive viral replication, characterized by the formation of multinucleated giant cells with progressive loss of cognitive, behavioral, and motor deficits, is likely to occur only after severe immunosuppression and breakdown of the BBB. The pathological events that eventually lead to the development of HIVD may be initiated outside the CNS and involve the process of monocyte activation and many important events associated with passage of activated cells across the BBB. Perivascular macrophages likely play a critical role in the pathogenesis of HIVD as they are located on the parenchymal side of the BBB and the pool is continuously renewed through bone marrow-derived macrophages, particularly during CNS inflammation [14]. Thus, the bone marrow may serve as a source of HIV-1-infected macrophages and may play a critical role in neuroinvasion and progression of CNS disease.

Genetic variation within the HIV-1 viral genome is a naturally occurring process driven by the low fidelity of reverse transcriptase, coupled with the selective pressures brought about within the host such as antiretroviral therapy, recreational drug use, immunological pressures, viral recombinatory events, host-cell phenotype, and rates of virus production [17-19]. These events result in single nucleotide polymorphisms (SNPs) throughout the genome including the promoter region, designated the long terminal repeat (LTR). Genetic variation occurs within LTR binding sites where host transcription factors and viral regulatory proteins bind, altering the way the LTR drives viral transcription. The resultant viral quasispecies are likely shaped by the selective pressures operative within a variety of cellular and tissue niches that ultimately maintain specific sets of quasispecies to form viral reservoirs in susceptible cell types and end-organ tissues [20-27]. The accumulation of specific LTR sequence configurations over time may also result from accumulation of poorly replicating viruses or latent proviruses in long-lived cell subsets in circulation or within viral reservoirs, such as the resting memory CD4^+^ T-cells, monocytes and macrophages, and hematopoietic progenitor cells (HPCs) [28].

Transcription factor binding sites within the LTR alternatively recruit both activating and repressing factors that can phenotypically alter the basal and inducible rates of viral gene expression [29-33]. This differential regulation may ultimately impact viral replication in both a cell type- and a tissue-specific manner [13,21,34,35] that may impact the course of HIV-1 disease [36]. Due to sequence variation within the LTR, *cis*-acting transcription factor binding sites in the LTR may be functionally altered during the evolution of quasispecies, resulting in altered promoter activity [20-22] and altered transcription factor binding [23,25-27]. Studies in the pre-HAART era have demonstrated that specific HIV-1 LTR CCAAT/enhancer binding protein (C/EBP) and Sp protein binding site configurations that arise as a consequence of quasispecies evolution, such as a C-to-T SNP at nucleotide position 3 in C/EBP site I within the context of a subtype B consensus sequence binding site (designated 3 T) and the C-to-T change at nucleotide position 5 in Sp binding site III (designated 5 T), were preferentially encountered in patients with more severe disease [23,37] and the brains of patients with HIVD [37]. Specific nucleotide changes within the LTR, such as the 3 T and 5 T, abrogate binding of cognate transcription factors to their corresponding binding site [23,25-27,37,38]. Variations in the Sp GC box array of the viral promoter result in altered rates of viral gene expression and viral replication [39-44]. In addition, in the HAART era the 5 T SNP in Sp site III has been found to occur almost as frequently as the consensus B (conB; derived January 2002 by the Los Alamos National Laboratory) for that site within the DrexelMed HIV/AIDS Genetic Analysis Cohort, which currently consists of approximately 500 HIV-1-infected patients (data not shown). Furthermore, in a small subset of those patients, the 3 T and 5 T SNPs occur together. Therefore, to better understand the significance of specific SNPs found in current and past HIV/AIDS cohorts with respect to promoter function, LTR activity was examined in the TF-1 progenitor, U-937 monocytic, and the Jurkat T-cell lines within a chromatin-based environment.

## Results

### Parental (wild-type) and 3T5T LTR results in cell type–specific phenotypes within different promoter backbones

The DrexelMed HIV/AIDS Genetic Analysis Cohort is currently comprised of approximately 504 patients, 78.26% of whom are on continuous highly active antiretroviral therapy (HAART). Within this patient cohort, 457 patients and 1,113 longitudinal samples have been genotyped and the HIV-1 LTRs contain many SNPs, including the previously identified C-to-T SNP at position 5 (5 T) in Sp site III, that occurs in approximately 24.9% of the LTRs analyzed to date compared to the conB sequence (GAGGCGTGGC) derived from the Los Alamos National Laboratory, which occurs at in about 67.8% of the LTRs analyzed. The 5 T SNP results in dramatically reduced binding of Sp1, Sp3, and the truncated form of Sp3 [23]. Approximately 5.1% of the patients within this cohort have a SNP within the LTR at C/EBP site I (AGCTTTCTACAA), where there is a C-to-T change at position 3 (3 T) that also results in a knockout binding phenotype [23,25-27,37,38]. Finally, a small number of the patients (1.0%) in the DrexelMed HIV/AIDS Genetic Analysis Cohort have been found to have the 3T5T double variant.

**Figure 1:**
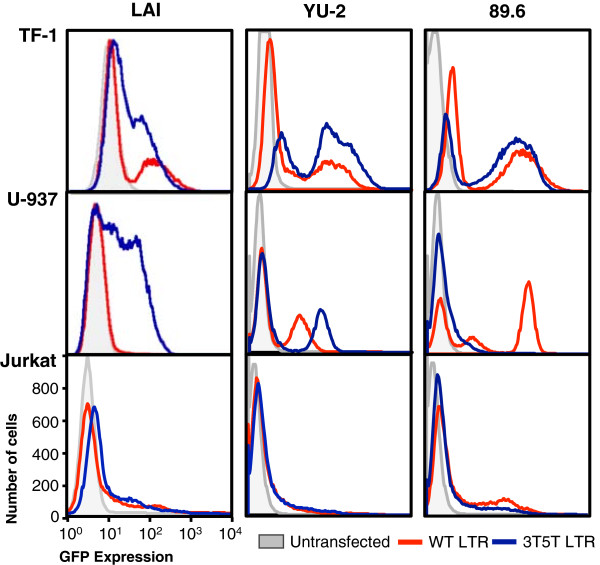
**Parental (wild-type; WT) and 3T5T long terminal repeat (LTR) results in cell type–specific phenotypes within different promoter backbones.** TF-1, U-937, and Jurkat cells were stably transfected with the HIV-1 LAI, YU-2, and 89.6 LTRs, which were placed in a green fluorescent protein expression vector (pEGFP-N1) using the AMAXA Nucleofector System (Lonza, Basel, Switzerland). Mutagenesis was used to introduce the position 3 C-to-T change at CCAAT/enhancer binding protein (C/EBP) site I and a position 5 C-to-T change at Sp site III (3T5T). Flow cytometric analysis was used to measure the ability of the variant LTR to drive GFP expression as compared with their parental (WT) counterparts. The fluorescence patterns in untransfected cells are depicted in gray. The LAI WT LTR is shown in red and the LAI 3T5T LTR is shown in blue.

As one approach to understand the effects of the 3 T and 5 T variations together on LTR-driven gene expression within several backbones, the WT and 3T5T variations were introduced into the LAI, YU-2, and 89.6 HIV-1 LTR backbones and then ligated into the pEGFP-N1 vector where the LTR can drive the expression of the green fluorescent protein (GFP). The constructs were then stably transfected into TF-1, U-937, and Jurkat cells. These cells were selected because they were all representative of cellular phenotypes that have been identified as potential or confirmed viral reservoirs where HIV-1 could potentially avoid recognition from immune cells and therapeutic agents, thus enabling the virus to survive for long periods within the host and to continue to replicate when the intra- and extracellular environment was conducive to virus production [2,13,28,33,45]. Within TF-1 cells, the 3T5T YU-2 and 89.6 LTRs resulted in both a nonexpressing population and a high-expressing population (Figure 1), whereas the 3T5T LAI LTR also resulted in an intermediate-expressing population. The intermediate-expressing population of cells was also observed within U-937 cells containing the 3T5T LAI LTR, whereas it was absent from U-937 cells containing the 3T5T YU-2 or 89.6 LTRs (Figure 1). In contrast, no phenotypic differences were found between the WT and 3T5T LAI, YU-2, or 89.6 LTRs within Jurkat cells (Figure 1), indicating that this cell phenotype may play in differentially controlling expression of LTRs containing genotypic alterations. The LAI backbone was selected for further study due to the presence of the large intermediate expressing cell population within cells containing the 3T5T LAI backbone that was mainly absent in both the YU-2 and 89.6 backbones (Figure 1). Additionally, it has been commonly used in other scientific studies, shares sequence similarity with the conB sequence except that it contains a 6G change within C/EBP site I, and the HIV-1 LAI virus has the ability to infect all three cell line models used in this study.

### Double-variant LTR results in two different phenotypes in hematopoietic progenitor, promonocytic, and Jurkat T cells

**Figure 2:**
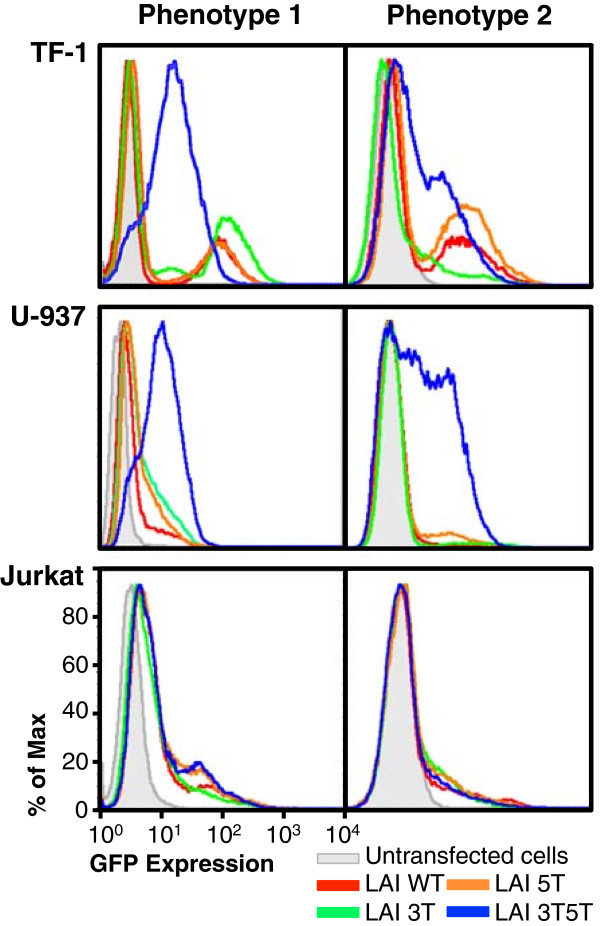
**Cell populations independently recovered from low-temperature storage resulted in two general phenotypes with respect to HIV-1 3T5T long terminal repeat–green fluorescent protein (LTR-GFP) basal expression.** TF-1, U-937, and Jurkat cells were stably transfected with the HIV-1 LAI LTR, which was placed in a GFP expression vector (pEGFP-N1) using the AMAXA Nucleofector System (Lonza, Basel, Switzerland). Mutagenesis was used to introduce the position 3 C-to-T change at CCAAT/enhancer binding protein (C/EBP) site I (3 T), a position 5 C-to-T change at Sp site III (5 T), or to introduce both the 3 T and 5 T variants into the same LAI LTR (3T5T). Flow cytometric analysis was used to measure the ability of each LTR to drive GFP expression as compared with their parental (WT) counterparts. The fluorescence patterns in untransfected cells are depicted in gray. The LAI WT LTR is shown in red; LAI 3 T LTR in green; LAI 5 T LTR in orange; and the HIV-1 LAI 3T5T LTR in blue. Phenotype expression pattern 1 is shown by panels on the left, while the phenotype expression pattern 2 is shown by panels one the right. All cells were analyzed by flow cytometry for basal levels of LTR-driven GFP expression between passages 6 and 8.

Transfected cells were selected under G418 selective pressure for over 4 weeks to develop stably transfected cell lines. Those cell lines were then frozen and preserved for future experimental use. As one approach to understanding the basic effects of genetic variation on LTR function, basal LTR-driven GFP expression was assessed using flow cytometry to examine the LTR-driven GFP expression patterns obtained from a number of cell populations independently recovered from low-temperature storage.Interestingly, each recovered cell population resulted in different LTR-driven expression patterns. This was particularly evident within the cell populations containing the 3T5T double-variant LTR, which could be grouped into two general phenotypic expression patterns. Stored frozen cells were independently recovered and grown in culture conditions more than six times each; representative histograms are shown in Figure 2. With TF-1 cells, generally phenotype 1 was characterized by a very small nonexpressing population of cells, in addition to a larger population of cells that express intermediate levels of LTR-driven GFP expression (Figure 2, top left). Within TF-1 cells, phenotype 2 was generally characterized by a large population of non-GFP-expressing cells as well as a population of cells that expressed both intermediate and high levels of GFP (Figure 2, top right). The TF-1 cell lines containing the HIV-1 parental LAI LTR as well as the 3 T and 5 T variant LTR generally exhibited the same two LTR-driven GFP expression phenotypes in that they exhibited large non-GFP-expressing populations of cells and a smaller high-GFP-expressing population of cells (Figure 2, top panels). The 5 T genotype generally resulted in higher amounts of LTR-driven GFP expression (Figure 2, top panels) when compared with GFP expression driven by the WT and 3 T-containing LAI LTR.Similar to the stably transfected TF-1 cells, basal LTR-driven GFP expression from stably transfected U-937 cell populations independently recovered from low-temperature storage also resulted in two distinct phenotypes specifically visible with the 3T5T-containing LTR cell populations (Figure 2, middle panels). In the U-937 cells, phenotype 1 was observed as a small non-GFP-expressing population with a larger intermediate-GFP-expressing population (Figure 2, middle left), whereas the 3T5T U-937 phenotype 2 cell population included a large non-GFP-expressing cell population and large populations of intermediate- and high-GFP-expressing cells (Figure 2, middle right). With respect to the U-937 cell populations carrying other LTR genotypes (WT, 3 T, and 5 T), the expression profiles consisted of mainly non/low levels of GFP expression and smaller populations of intermediate-GFP-expressing cells (Figure 2, middle panel). When the stably transfected Jurkat cells were recovered from low-temperature storage, they too displayed two distinct phenotypes. Phenotype 1 was characterized by a large low-expressing population of GFP-expressing cells within all Jurkat cell populations carrying each genotype and smaller populations of intermediate/high-GFP-expressing cells (Figure 1, bottom left). Within phenotype 2, there was a large population of nonexpressing cells that was similar to the WT Jurkat cell population, and a smaller population of cells that exhibited low/intermediate levels of GFP expression (Figure 2, bottom right).

### Double-variant LTR results in altered gene expression in basal and stimulated stably transfected cell lines

To better understand the kinetics behind LTR-driven gene transcription within LTRs that contained the SNPs of interest, the genotypic changes were analyzed in the presence of LTR activators such as tumor necrosis factor (TNF)-α and the viral transactivating protein Tat. For the following studies, TF-1, U-937, and Jurkat cell populations displaying phenotype 2 were selected for further study because of their large populations of non-GFP-expressing cells, which could represent a model cell line population representative of known cellular HIV-1 reservoirs within the body containing an integrated LTR structure in a non-expressing or silent mode that could be activated under the selected physiological conditions.

**Figure 3:**
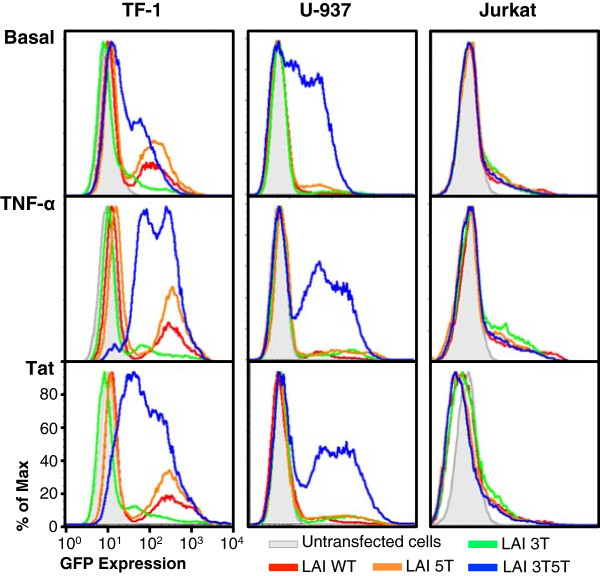
**Double-variant 3T5T long terminal repeat (LTR) results in altered gene expression in the stably transfected cell lines.** U-937 cells were stably transfected with the LAI LTR, cloned within the context of the green fluorescent protein expression vector pEGFP-N1 using the AMAXA Nucleofector System (Lonza, Basel, Switzerland). Mutagenesis was performed to introduce the position 3 C-to-T change at CCAAT/enhancer binding protein (C/EBP) site I (3 T), a position 5 C-to-T change at Sp site III (5 T), or to introduce both the 3 T and 5 T mutations into a single LTR (3T5T). Flow cytometric analysis was used to measure the ability of each LTR to drive GFP expression as compared with their parental (WT) counterparts. Untransfected cells are depicted in gray. The LAI WT LTR is shown in red; LAI 3 T LTR in green; LAI 5 T LTR in orange, and the LAI 3T5T LTR in blue. Under basal conditions, cells expressing the 3T5T LTR expressed an intermediate level of GFP when compared with WT and the other variants that mainly had low GFP expression. Stably transfected cells were treated with TNF-α (20 ng/mL) for 24 hours and then GFP expression was assessed using flow cytometry. The cells expressing the 3T5T LTR resulted in an increase in the high-GFP-expressing population. In addition, cells expressing GFP driven by the 3 T LTR showed a great increase in high-GFP-expressing cells. Stably transfected cells were transfected with Tat86 (300 ng) using the AMAXA Nucleofector System. Tat stimulation of the LTRs was shown to drive a very high level of GFP expression in the 3T5T LTR-GFP-expressing cells, and a small increase in intermediate/high-GFP-expressing cells when driven by the 3 T LTR.

Inflammatory cytokines such as TNF-α are elevated in patients with AIDS, and these particular cytokines are also involved in monocyte-macrophage differentiation as well as activation of HIV-1 gene transcription [46-53]. To study the effects of inflammatory cytokines on genetically altered LTR-driven gene expression in TF-1, U-937, and Jurkat cells, cells were treated with TNF-α (20 ng/mL) for 24 hours, after which time GFP expression was analyzed using flow cytometry. TNF-α stimulated cells of the monocytic and T-cell lineage through the binding of TNF-α to the TNF receptor, which causes the phosphorylation and release of IκB from nuclear factor-κB (NF-κB), in turn allowing NF-κB to translocate from the cytoplasm into the nucleus of the cell. NF-κB has then been shown to be able to activate many host and viral genes through the initial recruitment of the positive transcription elongation factor, as previously reviewed [45]. In addition, in the absence of functional Sp sites, NF-κB binding to the enhancer region of the LTR can result in alteration in viral replication in T cells [43-45,54]. As expected, the cells were stimulated by exposure to TNF-α, and as a result of the stimulation, LTR-driven gene expression was increased in each of the cell populations containing the selected LTR genotypes (Figure 3, middle row). In cells expressing the WT, 3 T, and 5 T LTRs, there was still a large population of cells that remained nonexpressers, while there was also a shift in the low-intermediate-expressing cells to a higher level of GFP expression (Figure 3). Interestingly, the 5 T LTR resulted in a higher level of change in LTR-driven GFP expression than the WT LTR, indicating that this genotype (which occurs frequently in the DrexelMed HIV/AIDS Genetic Analysis Cohort) could be involved in evading immune activation within the host under basal conditions, but under stimulatory conditions the 5 T genotype could be activated to produce a higher level of gene expression and virus production. Additionally, the stimulated 3T5T LTR exhibited a shift of the low-intermediate-GFP-expressing cell population to a majority of cells expressing a high level of GFP, and the majority of the nonexpressing cell population shifted to an intermediate level of GFP expression (Figure 3).Unexpectedly, the U-937 cells expressing GFP driven by the WT, 3 T, and 5 T LTRs were not stimulated to any great extent by TNF-α treatment. Normally, TNF-α would cause U-937 cells to differentiate from their promonocytic state into monocyte-macrophages, which would lead to an alteration in general cellular gene expression (Figure 3). In contrast, as a result of the stimulation, the 3T5T LTR-driven gene transcription and gene expression as modeled by GFP expression were elevated (Figure 3). In these cells, the population of nonexpressing cells showed no change in GFP expression; however, the lower-GFP-expressing cell population decreased in prevalence dramatically, resulting in a separate population of cells expressing intermediate/high levels of GFP as driven by the 3T5T LTR.

During infection with HIV-1, the virus primarily infects and replicates in activated CD4^+^ T cells. Despite the short lifespan of infected CD4^+^ T cells, a small fraction of infected, activated T cells return back to a resting memory state in which the virus no longer replicates. This phenomenon results in a stable, persistent viral infection that is transcriptionally silent until the cell is reactivated. The memory CD4^+^ T-cell has been the most widely studied latently infected cell phenotype, and these studies have provided extensive knowledge concerning how HIV-1 may remain latent in a cellular viral reservoir. The Jurkat T-cell line is comprised of quiescent, immortalized T-cells and provides an in vitro system that can model what occurs in inactivated T cells in an HIV-1-infected patient. To study the activity of the 3 T, 5 T, and 3T5T LTRs within the Jurkat cell line, stably transfected cells stimulated with TNF-α (20 ng/mL), and as a result the WT, 3 T, 5 T, and 3T5T LTRs displayed an increase in LTR-driven gene transcription (Figure 3). The LAI 3 T and 5 T LTRs exhibited a slightly higher level of gene transcription when stimulated, indicating that there might be a cell type-specific LTR activation with a specific genotype.

### Double-variant LTR results in more efficient binding of NF-κB complexes to the LTR

**Figure 4:**
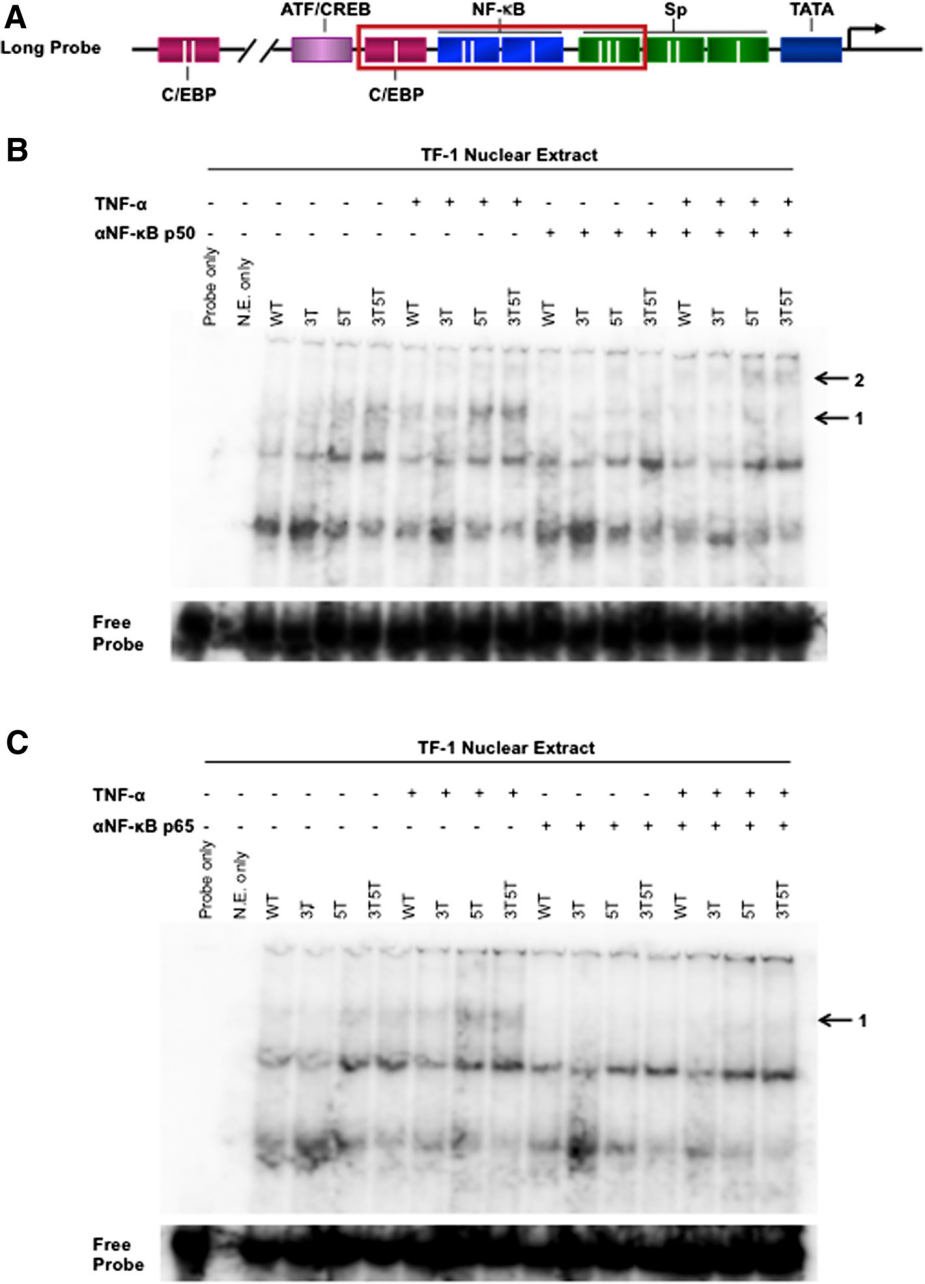
**Nuclear factor-κB (NF-κB) p50 and p65 bind more efficiently to the 5 T and 3T5T long terminal repeats (LTRs).** Nuclear extract was isolated from normal and tumor necrosis factor-α (TNF-α)-treated TF-1 cells. **(A)** An LAI long probe covering CCAAT/enhancer binding protein (C/EBP) site I, NF-κB site II, NF-κB site I, and Sp site III with the wild-type (WT), 3 T, 5 T, or 3T5T single-nucleotide polymorphisms (SNPs) was used to determine differences in complex formation at each LTR. TF-1 nuclear extract was incubated with probe in the absence or presence of **(B)** NF-κB p50 or **(C)** p65 gel shift antibodies for 30 minutes. **(B)** Normal and activated extracts incubated with the 5 T and 3T5T long probe showed greater complex formation and abrogation and shift of complex formation when incubated with NF-κB p50 antibody (black arrows). **(C)** Normal and activated extracts incubated with the 5 T and 3T5T long probe showed greater complex formation and abrogation of complex formation when incubated with NF-κB p65 antibody (black arrow).

As seen in Figures 2 and 3, the 5 T and 3T5T SNPs resulted in altered LTR-driven GFP expression. To determine whether the 5 T and 3T5T SNPs altered binding of transcription factors to their cognate binding sites on the LTR, and whether the 3T5T SNP could potentially lead to altered and more efficient binding of NF-κB complexes to NF-κB site I and site II, electromobility shift assays were performed. Nuclear extract was isolated from WT untransfected TF-1 cells that were untreated or treated with 20 ng/mL TNF-α for 24 hours and then incubated with the long probes containing the WT, 3 T, 5 T, and 3T5T variants depicted in Figure 4A. Extracts were also incubated in the absence or presence of gel shift antibodies against NF-κB p50 and p65. When extracts were incubated with the 5 T and 3T5T long probe, in both activated and nonactivated conditions, a large molecular weight complex (arrow 1 in Figure 4B) formed more prominently with those two probes in comparison to the others. Additionally, incubation with the NF-κB p50 antibody resulted in abrogation of binding of that complex to the long probe, and a shift in activated extracts incubated with the 5 T and 3T5T probes (arrow 2 in Figure 4B). This observation indicates that 5 T and 3T5T configurations allow for better binding of NF-κB p50 to its cognate binding site, potentially due to the lack of steric hindrance from both C/EBP and Sp factors. When extracts were incubated with long probes and the NF-κB p65 antibody, again, the same high molecular weight complex was more abundant with the 5 T and 3T5T probes, and binding of that complex to all probes was abrogated with the NF-κB p65 antibody (arrow in Figure 4C). The inability of C/EBP and Sp factors to bind efficiently to the mutated binding sites probably leads to the more efficient binding of NF-κB factors. These results also indicate that under activated conditions, that there is a larger complex loading to the 5 T- and 3T5T-containing LTRs and that it is comprised of NF-κB p50 and p65, suggesting that under normal conditions, the loading is not as effective. This could explain the intermediate levels of expression observed in Figure 3, and under activated conditions the much higher level of activation that occurs with those genotypes.

### Stably transfected cell clones from TF-1 and U-937 cell lines reflect the GFP expression profiles of the total populations used to select the cell clones

**Figure 5:**
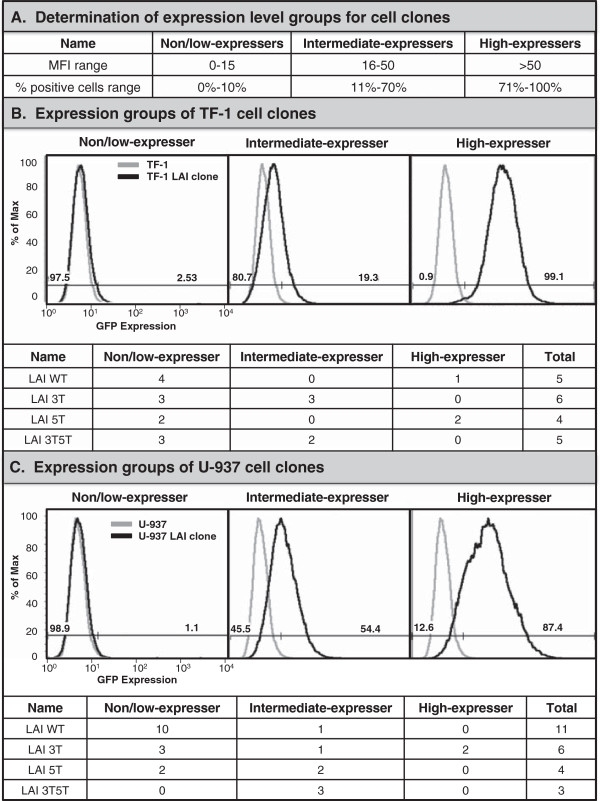
**Cell clones developed from total populations of stably transfected TF-1 and U-937 cell lines reflect the green fluorescent protein (GFP) expression profile of the parental populations.** The TF-1 and U-937 cells that were stably transfected with the LAI long terminal repeats (LTR) (wild-type [WT], 3 T, 5 T, and 3T5T) were serially diluted in order to obtain 1 cell in 1 mL of media (approximately 1 cell in 10 wells of a 96-well plate). Cell clone populations were propagated from a single cell and then were analyzed using flow cytometry for their basal GFP expression. The clonal populations were then designated in one of three categories (nonexpresser, intermediate expresser, and high expresser) based on their geometric mean fluorescence intensity (MFI) and their percent cell positive values **(A)**. Representative histograms showing levels of GFP expression by the stably transfected cell clone (black line) compared with the untransfected control cell line (gray line) for TF-1 **(B)**, U-937 **(C)**, cell clones expressing the LAI WT, 3 T, 5 T, or 3T5T LTRs. The histograms show that the TF-1 and U-937 cell clones mimic their parental population counterparts in terms of basal GFP expression profiles. Tables below each set of histograms show the total number of clonal cell populations for each LTR genotype that falls within one of the three expression phenotypes.

In order to effectively study how the LTR functions to drive GFP expression from the standpoint of a single cell, sets of clones were developed from each stably transfected cell line (TF-1 and U-937) using serial dilution and then propagating a population from a single cell. Once each single cell gave rise to a population of clones, their basal level of LTR-driven GFP expression was assessed by flow cytometry. Each clonal population was designated into one of three categories: nonexpresser, intermediate expresser, or high expresser based on arbitrary designations from the geometric mean fluorescence intensity (MFI) and GFP-positive cell percentage (Figure 5A). Figure 5B displays representative histograms for stably transfected TF-1 clonal populations containing the WT, 3 T, 5 T, or 3T5T LTR that fall into each category. There are a total of four nonexpresser and one high-expresser TF-1 LAI LTR WT clones; three nonexpressing and three intermediate-expressing TF-1 LAI LTR 3 T clones; two nonexpressing and two high-expressing TF-1 LAI LTR 5 T clones; and three nonexpressing and two intermediate-expressing TF-1 LAI LTR 3T5T clones (Figure 5B). When these clones were compared with the populations from which they were derived (Figure 3, top panel), their expression profiles are representative of the variant cell line from the expression pattern observed under basal conditions prior to cell cloning. Figure 5C displays representative histograms for stably transfected U-937 promonocytic clonal populations containing the WT, 3 T, 5 T, or 3T5T LTR that fall into each category. There are a total of 10 nonexpressing and one intermediate-expressing U-937 LAI LTR WT clones; three nonexpressing, one intermediate expressing, and two high-expressing U-937 LAI LTR 3 T clones; two nonexpressing and two intermediate-expressing U-937 LAI LTR 5 T clones; and three intermediate-expressing U-937 LAI LTR 3T5T clones (Figure 5C). Interestingly, the Jurkat T-cell clones were difficult to propagate because as single cells, they would become quiescent and had a propensity to die quickly instead of multiplying. Therefore it was difficult to obtain a large number of Jurkat T-cell clones to study. Additionally, because of the low LTR-driven GFP activation within the Jurkat T-cell populations, they were no longer used for the purposes of this study.

### Nonexpressing 3T5T LAI LTR within TF-1 cells can be induced to drive GFP expression

**Figure 6:**
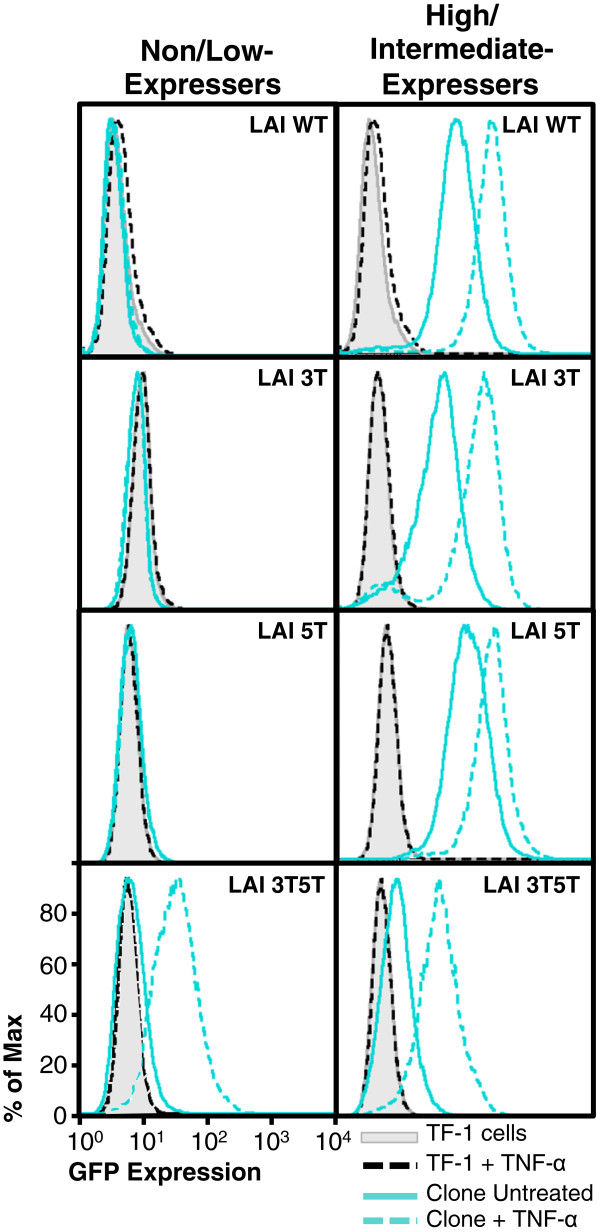
**Nonexpressing TF-1 cell clones containing the 3T5T LAI long terminal repeat (LTR) could be induced into LTR-driven green fluorescent protein (GFP) expression.** TF-1 cells stably transfected with the HIV-1 LAI LTR (wild type [WT], 3 T, 5 T, and 3T5T) were serially diluted in order to obtain 1 cell in 1 mL of media (approximately 1 cell in 10 wells of a 96-well plate). Cell clone populations were propagated from the single cell and then were analyzed using flow cytometry for their basal GFP expression. The clonal populations were then designated in one of three categories (nonexpresser, intermediate expresser, and high expresser) based on their geometric mean fluorescence intensity (MFI) and their percent cell positive values (Figure 5A). Non/low-expressing and high-expressing LAI WT and LAI 5 T LTR containing clones were treated with a range of tumor necrosis factor-α (TNF-α) concentrations (20–300 ng/mL). Representative histograms (at a TNF-α concentration of 20 ng/mL) showing levels of GFP expression obtained with the untreated, stably transfected cell clone (solid turquoise line) compared with the treated, stably transfected cell clone (dashed turquoise line), untreated WT TF-1 cells (solid black line), and treated WT TF-1 cells (dashed black line). Each clone shown is a representative of the group.

Because of the expression phenotypes observed with the nonexpressers and intermediate expressers, it was of interest to determine whether stimulation of the LTR with inflammatory cytokines could induce these LTRs to drive GFP expression. If so, this might represent a model for “promoter latency and reactivation” within the bone marrow progenitor cell type with LTRs containing naturally occurring SNPs. TF-1 cell clones were treated with TNF-α (at 20, 50, 100, 200, or 300 ng/mL) to determine whether inflammatory cytokine induction of the LTR could drive GFP expression in these cells. Clones that had higher levels of GFP expression were used as controls to show that the LTR was inducible through treatment. The titration of TNF-α concentrations resulted in very similar levels of LTR-induced GFP expression (data not shown). Therefore, in this and subsequent experiments, only representative data showing the results of the TNF-α treatment at 20-ng/mL were shown (Figure 6). Under stimulated conditions, the high-expressing TF-1 clones containing the LAI WT or 5 T LTR drove a higher level of GFP expression than the same unstimulated LTRs (Figure 6). In contrast, under the same conditions, the TF-1 nonexpressing clones containing the LAI WT or 5 T LTRs were not stimulated to drive GFP expression. Stimulated nonexpressing TF-1 clones containing the LAI 3 T LTR were not induced into driving higher levels of GFP expression; however, the intermediate-expressing clone used as a control was induced and was thereby able to drive a higher level of GFP expression. Interestingly, the difference in MFI values between stimulated and unstimulated GFP levels of expression was greater with the clone containing the 3 T variant than the difference between stimulated and unstimulated MFI observed with the WT and 5 T LTRs. As shown in Figure 5B, the 3T5T LAI LTR resulted in both a population and clones that had intermediate levels of LTR-driven GFP expression. Therefore, for this experiment, a low-expressing clone and an intermediate-expressing clone were selected to represent non- and high-expressing clones, respectively. Under stimulated conditions, the low- and intermediate-expressing LTRs drove a higher level of GFP expression than in unstimulated conditions within the TF-1 cell background. However, the low-expressing 3T5T cell clone had a larger difference in MFI between unstimulated and stimulated conditions when compared with the intermediate-expressing cell clone (Figure 6).

### U-937 clones containing nonexpressing LTR phenotypes cannot be induced into driving GFP expression

**Figure 7:**
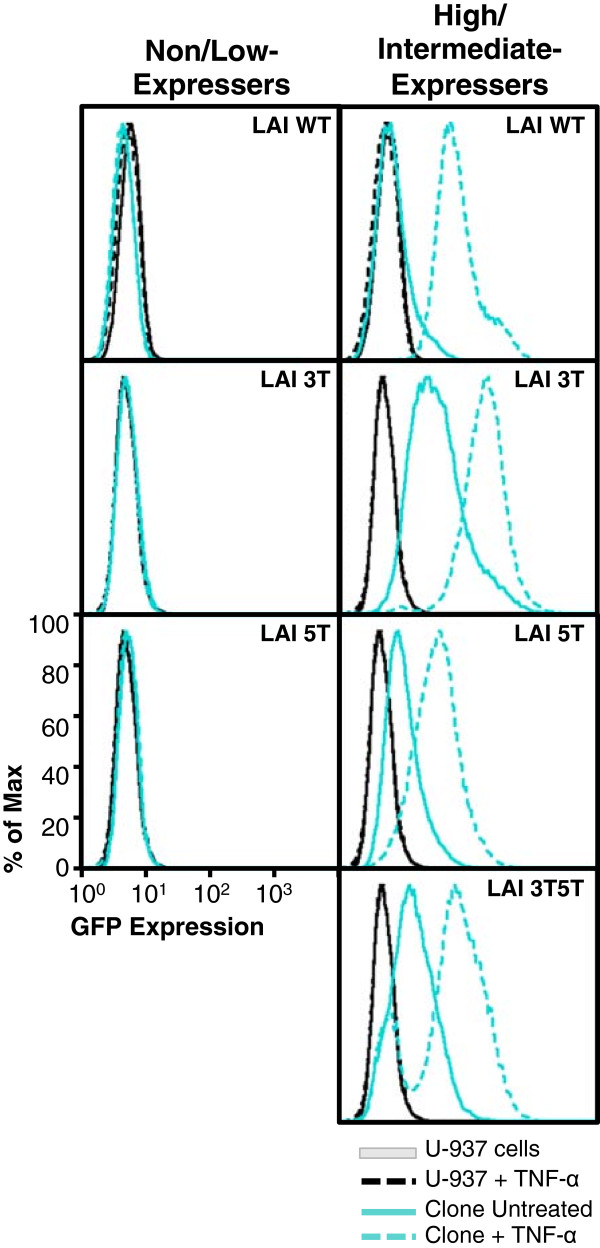
**U-937 cell clones containing nonexpressing wild-type (WT) and variant long terminal repeats (LTRs) cannot be induced into driving green fluorescent protein (GFP) expression.** U-937 cells stably transfected with the LAI LTR (WT, 3 T, 5 T, and 3T5T) were serially diluted in order to obtain 1 cell in 1 mL of media (approximately 1 cell in 10 wells of a 96-well plate). Cell clone populations were propagated from the single cell and then were analyzed using flow cytometry for their basal GFP expression. The clonal populations were then designated in one of three categories (nonexpresser, intermediate expresser, and high expresser) based on their geometric mean fluorescence intensity (MFI) and their percent cell positive values (Figure 5A). Nonexpressing and expressing cell clones were treated with a range of tumor necrosis factor-α (TNF-α) concentrations (20–300 ng/mL). Representative histograms (at a TNF-α concentration of 20 ng/mL) showing levels of GFP expression obtained with the untreated, stably transfected cell clone (solid turquoise line) compared with the treated, stably transfected cell clone (dashed turquoise line), untreated WT U-937 cells (solid black line), and treated WT U-937 cells (dashed black line). As there were no nonexpressing U-937 3T5T LTRs containing clones, they are excluded from the non/low-expressing cell clone column. Each clone shown is a representative of the group.

To determine whether the non/low-GFP-expressing phenotypes observed within the U-937 cell clones could be induced into expression, unlike the stably transfected TF-1 cell clones, each of the cell clones was treated with TNF-α (20 ng/mL) for 24 hours and then analyzed for GFP expression using flow cytometry. Figure 7 shows representative histograms from both nonexpressing and expressing phenotypes within each backbone expressed within the stably transfected U-937 cell clones. With TNF-α stimulation, the high-expressing cell clone containing the LAI 3 T LTR drove a higher level of GFP expression than the same unstimulated LTR (Figure 7). In contrast, under the same conditions, the nonexpressing clone containing the LAI 3 T LTR was not stimulated to drive GFP expression. Stimulated nonexpressing clones containing the LAI WT and 5 T LTRs were also not induced into driving higher levels of GFP expression; however, the intermediate-expressing clones that were used as controls for each backbone were able to be induced and drive higher levels of GFP expression. Interestingly, the low-intermediate-expressing phenotype within the U-937 LAI WT cell clone had the largest difference in unstimulated versus stimulated MFIs (33.4 and 80.7, respectively) and percent positive GFP-expressing cell number (9.67% and 98.5%, respectively). As shown in Figure 5C and as one might expect, the 3T5T LAI LTR resulted in clones that had varying intermediate levels of LTR-driven GFP expression. Therefore, for this experiment, only one intermediate-expressing clone was selected to represent a high-expressing phenotype (Figure 7, bottom panels). Under stimulated conditions, the intermediate clone exhibited a transition into high-GFP-expressing cells, in comparison to the stimulated levels of the expressing clones containing the WT, 3 T, or 5 T LTRs.

## Discussion

Genetic variation within the HIV-1 genome is a naturally occurring phenomenon because of the low fidelity of reverse transcriptase as well as selective pressures present within the host [17,18]. These viral quasispecies are potentially able to establish niches and reservoirs within HPCs, cells of the monocyte-macrophage lineage, as well as T cells that can reseed infection into the periphery and CNS of infected patients and possibly other end organs as well [20-28]. Nucleotide changes within the NF-κB-proximal C/EBP and Sp transcription factor binding sites within the viral promoter have been observed in both the pre-HAART and HAART eras and have been shown to guide differential regulation of viral expression by the LTR [20-22,55-57]. Interestingly, the prevalence of the 3 T, 5 T, and 3T5T LTR variants in the HAART era, at least in the DrexelMed HIV/AIDS Genetic Analysis Cohort is lower when compared to the levels observed from published studies in the pre-HARRT era [23-27,37]. However, the DrexelMed HIV/AIDS Genetic Analysis Cohort is overall much healthier because of the prolonged use of combination antiretroviral therapy. However, we hypothesize that the prevalence of the 3 T, 5 T, and 3T5T LTR variants will become more prevalent with the development of greater disease severity across this HIV-1-infected patient population. In fact, one of the patients that has shown severe CD4 decline and neurologic impairment carries both of these variations as we have previously published [19].

The TF-1, U-937, and Jurkat T-cell lines were used as models of HPCs, monocytes, and CD4^+^ T cells, respectively, in order to study the effects of the previously described SNPs on LTR-driven gene expression within a chromatin-based microenvironment. Each cell type was stably transfected with a specific LTR variant of interest and then stored at low temperature for prolonged periods prior to experimentation. Flow cytometric analysis showed that subsequent recovery of the stored cell populations containing the HIV-1 variant LTR resulted in two different LTR-driven GFP expression phenotypes with each model cell line (TF-1, U-937, and Jurkat) and parental or variant LTR (3 T, 5 T, and 3T5T) (Figure 2). We theorized that once multiple cell samples were stored at low temperature for prolonged periods, the same expression phenotype would be maintained for each sample that was recovered from storage and passaged to approximately the same level. However, as shown in Figure 2, this was not the case; several phenotypes were associated with the specific genotypic changes within the LTRs stably transfected across all model cell lines. Alterations in gene expression from cells recovered from low-temperature storage could be caused by differences in the stably transfected cells that survive the thaw and reculturing process. Cell death is a major problem when the cell line populations are thawed and recultured, as cells with different phenotypes may be the ones surviving each time this cycle occurs. The cell clones that have very distinct expression profiles, and maintain their expression profiles during subsequent thaws and reculture from low-temperature storage, provide further evidence of this phenomenon. In another sense, a recent study showed that when rheumatoid arthritis synovial fibroblast (RASF) cells were in culture, as the culture passages increased from 2 to 8, up to 10% of the genes within this cell line are differentially expressed [58]. In contrast to our observations, however, after long-term storage, gene expression within the RASF after passage 2 was comparable to gene expression prior to freezing [58]. This is the first study to show that long-term storage of stably transfected cells of the HPC, monocytic, and T-cell lineages can result in alterations in gene expression patterns.

TNF-α treatment of each of the stably transfected cell line populations was performed to determine how each SNP of interest might impact the way the LTR drives GFP expression when observed from cell populations recovered from long-term low- temperature storage. Stably transfected U-937 cells containing the 3 T and 3T5T LTRs exhibited 2.34- and 2.55-fold increases in MFI, respectively, with TNF-α stimulation. The difference in LTR-driven gene transcription with LTRs containing the WT, 3 T, 5 T, and 3T5T SNPs between stably transfected TF-1 and U-937 cells could be attributed to the difference in cell types and the availability of specific transcription factors and inherent viral gene activities within those cells. Within the stably transfected TF-1 cells treated with TNF-α, the 5 T LTR resulted in a higher level of change in LTR-driven GFP expression than the WT LTR (Figure 3). This observation indicates that although this genotype (which occurs frequently in the DrexelMed HIV/AIDS Genetic Analysis Cohort) could be involved in evading immune activation within the host under basal conditions, under stimulatory conditions it might be activated to produce a higher level of gene expression and virus production. Additionally, TNF-α stimulation of the stably transfected TF-1 and U-937 cells (Figure 3) suggested that a 3T5T LTR-containing proviral DNA could remain quiescent under certain metabolic conditions or in a basal viral gene expression mode. Only minimal intracellular stimulatory conditions would be required to drive low levels of viral gene expression and virus production within these cell populations, allowing evasion of HAART elimination of the infecting cell. The viral genome containing the 3T5T LTR could promote the evasion of effective HAART elimination, potentially contributing to the development of a niched viral reservoir for virus with specific genetic signatures that favor the quiescent or low-level gene expression mode. Subsequently, when an infected patient has a proinflammatory response to either the HIV-1 infection or an opportunistic infection, the 3T5T double-variant LTR-containing viral genotype could again result in an activation of the viral genome from a latent viral reservoir. This conclusion has been based on the increase in NF-κB binding to the 3T5T double variant and enhanced operation of these low-affinity C/EBP and Sp binding sites in the presence of elevated levels of the corresponding activation factors and a repopulation of circulating virus within the patient’s peripheral blood and lymphoid tissues and within specialized reservoirs. These changes would thereby make HAART less effective in removing and reducing virus from within the immune and nervous systems as well as other end organs.

The cell clones from each stably transfected cell line containing the WT, 3 T, 5 T, or 3T5T LTRs were developed to facilitate studies to explain the observable differences between each of the LTRs within each cell line. The low-GFP-expressing 3T5T LTR-containing TF-1 cell clone exhibited a larger difference in MFI between unstimulated and stimulated conditions compared with the intermediate-GFP-expressing cell clone. This observation may suggest that virus containing the 3T5T LTR genotype with a low enough expression profile in the body to facilitate evasion of HAART and the immune response, could, under stimulated conditions, produce a large amount of virus, reseeding infection into the body, possibly with viral strains that may be more resistant to treatment. These observations suggest that across LTRs containing each of the SNPs of interest (3 T, 5 T, and 3T5T), there is a differential expression within individual GFP-expressing clones. These results also show that within an LTR, in this case the 3T5T-containing LAI LTR, stimulation by cytokines naturally occurring during inflammation and infection can lead to differential regulation of the LTR between different cell lines. In addition, non-GFP-expressing cell clones, regardless of the LTR or cell type, could not be induced into expression with TNF-α stimulation, whereas the intermediate- and high-GFP-expressing cell clones containing each SNP of interest could be induced. This important result shows that differences in regulation of LTR-driven expression may also be attributable to the location of integration within the human genome, proviral DNA copy number, or possibly differences in epigenetic controls operative on the LTRs.

## Conclusions

The experimental approach used in this study has provided a means for understanding SNP-specific LTR function within models of HIV-1-susceptible cells. However, this approach has not been without its limitations. In the model system used in this study, the function of the LTR is only observed within the context of an integrated plasmid. In addition, only the LAI backbone is used, which has been derived from an X4-tropic virus. The LAI backbone was used because (1) it has been commonly used in other scientific studies, (2) across the 12 binding sites within the LTR, it shares sequence similarity with the conB sequence except that it contains a 6G change within C/EBP site I, and (3) full-length HIV-1 LAI virus has the ability to infect all three cell line models used in this study. It would be interesting to determine the effects of the other LTR backbones, such as YU-2, an R5 virus, and 89.6, which has been defined to be an X4/R5 virus, on SNP-specific LTR driven gene expression within stably transfected TF-1, U-937, and Jurkat cells. It would also be important to determine whether the effects of these SNPs on LTR-driven gene expression observed within the stably transfected cells used in this study would remain if full-length pseudotype virus developed from molecular clones using the different HIV-1 backbones were used to infect the HPCs, monocytes, and T cells.

We have shown that the 3 T SNP within C/EBP binding site I and the 5 T SNP within Sp binding site III, both previously reported to correlate with differential binding of the C/EBP and Sp transcription factors for their cognate binding sites [23,25-27,43,44], cause differential regulation of gene expression by the LTR. We also showed that the combination of the two SNPs leads to low levels of LTR-driven GFP expression. The low levels of the double variant LTR-driven GFP expression could translate to a virus with a selective advantage, enhancing viral survival and facilitate the evasion of HAART therapy as well as the host immune response to viral infection. Under activating physiological conditions in the host, selected viral quasispecies of low fitness under basal conditions with appropriate *cis*-acting LTR elements could retain the ability to respond to activating stimuli and replicate at higher levels. They could thereby reseed infection into numerous compartments, once again expanding the quantity of virus and spectrum of viral quasispecies in the periphery which could potentially cross the BBB reseeding virus in the CNS.

## Methods

### Cell culture

The TF-1 CD34^+^ erythromyeloid leukemia cell line (ATCC, Manassas, VA) was grown in RPMI 1640 medium with l-glutamine (Cellgro, Herndon, VA) supplemented with 10% heat-inactivated fetal bovine serum (FBS; GemCell, West Sacramento, CA), antibiotics (penicillin and streptomycin, at a concentration of 0.04 mg/mL each; Cellgro), glucose (4.5 g/mL; Cellgro), sodium pyruvate (1 mM; Cellgro) and HEPES (10 mM; Cellgro), and recombinant human granulocyte-macrophage colony stimulating factor (2 ng/mL; eBioscience, San Diego, CA). The cells were maintained at a density between 1–5 × 10^5^ cells/mL.

The U-937 human promonocytic cell line (ATCC, CRL-1593.2) was grown in RPMI 1640 medium with l-glutamine supplemented with 10% heat-inactivated FBS, antibiotics (penicillin and streptomycin, at a concentration of 0.04 mg/mL each), glucose (4.5 g/mL), sodium pyruvate (1 mM), and HEPES (10 mM). The cells were maintained at a density between 1–5 × 10^5^ cells/mL.

The human T-cell line Jurkat (ATCC, TIB-152) was grown in RPMI 1640 medium with l-glutamine supplemented with 10% heat-inactivated FBS, sodium bicarbonate (0.05%), and antibiotics (penicillin, streptomycin, and kanamycin at 0.04 mg/mL each). The cells were maintained at a density between 1 × 10^5^ and 1 × 10^6^ cells/mL as recommended by ATCC. All cells were maintained at 37°C in 5% CO_2_ at 90% relative humidity.

### Plasmid construction and site-directed mutagenesis

The HIV-1 LAI LTR sequence was amplified by PCR from previously published LAI-LTR-Luc reporter constructs [59,60]. The following primers were used for the amplification of LAI LTR: 5′-GGC-CGG-ATT-AAT-TGG-AAG -GGC-TAA-TTC-ACT-CC-3′ and 5′-GAC-CGG-TTG-CTA-GAG-ATT-TTC-CAC-AC T-G-3′ (Integrated DNA Technologies). Following amplification, the HIV-1 LAI LTR was cloned into the pEGFP-N1 vector (Clontech, Mountain View, CA), which contains the eGFP gene. The PCR product and the pEGFP-N1 vector were digested with the *Vsp*I (*Ase*I) and the *Bsh*TI (*Age*I) restriction enzymes and the digested PCR product was ligated into the digested pEGFP-N1 vector, generating the LAI LTR-GFP reporter construct.

Site-directed mutagenesis (SDM) was performed using the QuickChange mutagenesis procedure (Stratagene, La Jolla, CA) on the HIV-1 LAI LTR-GFP reporter construct to incorporate a C-to-T mutation at position 5 of the Sp site III (5 T). The following primers were used: CTT TCC AGG GAG G**T**G TGG CCT GGG CG and CGC CCA GGC CAC **A**CC TCC CTG GAA AG. The mutagenized nucleotide is underlined. This process generated the LAI LTR-5 T-GFP reporter construct. SDM was also performed on the HIV-1 LAI LTR-GFP reporter construct to incorporate a C-to-T mutation at position 3 of the C/EBP site I (3 T). The following primers were used: GCT GAC ATC GAG **T**TT GCT ACA AGG G and CCC TTG TAG CAA **A**CT CGA TGT CAG C. The mutagenized nucleotide is underlined. This process generated the LAI LTR-3 T-GFP, which was used for an additional round of SDM to incorporate a C-to-T mutation at position 5 of the Sp site III (5 T) as indicated previously. This process generated the LAI LTR-3T5T-GFP reporter constructs. For both the parental and mutated HIV-1 LTR constructs authenticity was confirmed by DNA sequencing (Genewiz, South Plainfield, NJ) and sequence analyses using Lasergene software (DNASTAR, Inc., Madison, WI).

### Stable transfection of TF-1, U-937, and Jurkat cells and stably transfected cell clone development

TF-1, U-937, and Jurkat cells (ATCC) were transfected with the constructs using the AMAXA Nucleofector System and AMAXA procedure V, as described by the manufacturer (Lonza, Basel, Switzerland). Transfected cells were passaged under G418 (800 ng/mL; Mediatech, Manassas, VA) selection beginning 24 hours after transfection and passaged under G418 selection for at least 2 months to develop stably transfected populations. The TF-1 and U-937 cell populations were each serially diluted to a final concentration of 1 cell/mL media and plated into 96-well plates, where they were marked and tracked for growth. Once clonal cells became a confluent clonal population within the well, they were moved to a larger well with more media until they could be transferred to flasks and propagated normally. Basal levels of LTR-driven GFP expression within each cell population and clonal population were measured using flow cytometry.

### Assessment of GFP expression utilizing flow cytometry

TF-1, U-937, and Jurkat cells were washed with flow cytometry buffer [Hanks balanced saline solution (Mediatech), FBS (3%), and NaN_3_ (0.02%)] and aliquots of 1 × 10^6^ cells were fixed with paraformaldehyde (1%). Flow cytometry analysis was performed utilizing a Calibur Flow Cytometer (BD Biosciences, San Diego, CA) and analyzed the results using Flowjo version 8.8.7 software (Tree Star, Ashland, OR).

### Oligonucleotide synthesis and radiolabeling

Complementary single-stranded oligonucleotides corresponding to the LAI LTR with and without the 3 T SNP in C/EBP site I and/or the 5 T SNP in Sp site III were synthesized (Integrated DNA Technologies) and annealed by brief heating at 100°C followed by slow cooling to room temperature as previously described [37]. Blunt-ended double-stranded oligonucleotides were end-labeled using ^32^P-ATP and T4 polynucleotide kinase as described (Promega, Madison, WI). The sequences of the probes used in this study are as follows: LAI LTR WT: TCG AGC TTG CTA CAA GGG ACT TTC CGC TGG GGA CTT TCC AGG GAG GCG TGG CC; LAI LTR 3 T: TCG AG**T** TTG CTA CAA GGG ACT TTC CGC TGG GGA CTT TCC AGG GAG GCG TGG CC; LAI LTR 5 T: TCG AGC TTG CTA CAA GGG ACT TTC CGC TGG GGA CTT TCC AGG GAG G**T**G TGG CC; LAI LTR 3T5T: TCG AG**T** TTG CTA CAA GGG ACT TTC CGC TGG GGA CTT TCC AGG GAG G**T**G TGG CC. The SNPs are indicated as underlined nucleotides.

### Electromobility shift analysis

Electrophoretic mobility shift assay binding reactions were performed as previously described [37]. The radiolabeled LAI LTR probes were incubated with activated [TNF-α (20 ng/mL) for 24 hours] or normal nuclear extract from TF-1 cells for 30 minutes at 4°C in the presence of with poly[dI-dC)] (1 μg), bovine serum albumin (BSA), and 1× binding buffer. Reactions (20 μL, final volume) were supplemented with BSA (15 μg). The following antibodies (Santa Cruz Biotechnology, Santa Cruz, CA) were used to determine complex formation: NF-κB p50 (H-119X), NF-κB p65 (C-20X). DNA-protein complexes were resolved at 4°C by electrophoresis for 3 hours at 200 V in a 4.5% nondenaturing polyacrylamide gel, which was then dried at 80°C for 2.5 hours and used for autoradiography.

### Nuclear protein extraction from TF-1, U-937, and Jurkat cells

Nuclear protein extraction was performed as previously described [61]. Briefly, 3 × 10^7^ cells were harvested for each nuclear extraction and washed once with 1× phosphate-buffered saline. The cells were incubated for 15 minutes with lysis buffer on ice and then 2 μL 10% NP-40 detergent was added and cells were inverted several times. Cells were centrifuged at 1100 RPM for 5 minutes and then resuspended, through inverting, in lysis buffer and then centrifuged again. The nuclei pellets were then resuspended in nuclear extract buffer and then incubated for at 4°C for 30 minutes on a vortexer set to a speed of 3. The nuclear lysate was centrifuged at 14,000 RPM for 10 minutes at 4°C, after which time the supernatant was removed and quantitated with respect to protein concentration as previously described [62]. Nuclear extracts were then stored at −80°C.

## Abbreviations

BBB: Blood–brain barrier; BSA: Bovine serum albumin; C/EBP: CCAAT/enhancer binding protein; CNS: Central nervous system; FBS: Fetal bovine serum; GFP: Green fluorescent protein; HAART: Highly active antiretroviral therapy; HIVD: HIV-associated dementia; HPC: Hematopoietic progenitor cell; LTR: Long terminal repeat; MFI: Mean fluorescent intensity; NF-κB: Nuclear factor-κB; RASF: Rheumatoid arthritis synovial fibroblast; SNP: Single-nucleotide polymorphism; TNF-α: Tumor necrosis factor-α; WT: Wild type.

## Competing interests

The authors declare that they have no competing interests.

## Authors’ contributions

SS contributed to the development of stably transfected cell lines, developed the stably transfected cell clones, completed all flow cytometric acquisition and analysis, contributed to study conception and design, drafted figures, and drafted the manuscript. KA constructed the LTR-plasmid constructs and contributed to the development of stably transfected populations. SD contributed to the design and completion of the electrophoretic mobility shift assays. VP helped draft the manuscript. SS, MN and BW conceived of the study and participated in its design and coordination and helped to draft the manuscript. All authors read and approved the final manuscript.
